# miR-143 mediates abiraterone acetate resistance by regulating the JNK/Bcl-2 signaling pathway in prostate cancer

**DOI:** 10.7150/jca.78246

**Published:** 2022-11-21

**Authors:** Yigeng Feng, Hongwen Cao, Wenyang Zhao, Lei Chen, Dan Wang, Renjie Gao

**Affiliations:** Surgical Department I (Urology Department), LONGHUA Hospital Shanghai University of Traditional Chinese Medicine, No. 725 Wanping Road South, Xuhui District, Shanghai 200032, China

**Keywords:** miR-143, prostate cancer, abiraterone acetate, JNK/Bcl-2 signaling, drug resistance

## Abstract

**Background:** miR-143 is known to be downregulated in various cancer cells and tumors and generally plays a tumor-suppressor role. miR-143. However, the role of miR-143 in the mediation of the sensitivity of prostate cancer cells to abiraterone acetate remains unrevealed.

**Methods:** The expression levels of miRNAs were determined by miRNA microarray and quantitative real-time PCR (qRT-PCR). The protein levels were assessed by Western blot assay. Cell viability and apoptosis were respectively measured by Cell Counting Kit-8 (CCK-8) assay and flow cytometry.

**Results:** We identified that miR-143 was significantly downregulated in PC3-AbiR cells compared to PC3 cells. Overexpression of miR-143 promoted PC-AbiR sensitivity to abiraterone acetate in vitro and in vivo. We also revealed that miR-143 upregulation inhibited p-JNK (c-Jun N-terminal kinases) and increased p-Bcl2 (B-cell lymphoma 2), contributing to abiraterone acetate-induced apoptosis in PC3-AbiR cells. Finally, we showed that the combination of miR-143 and abiraterone acetate exerted the most profound tumor inhibition effect and prolonged the mice survival rate in PC3-AbiR tumor-bearing mice.

**Conclusion:** Upregulation of miR-143 may serve as a new strategy to enhance the therapeutical effect of abiraterone acetate on prostate cancer patients who are resistant to abiraterone acetate.

## Introduction

Prostate cancer is the most common solid tumor and is the second cause of cancer mortality in men 1. The incidence of prostate cancer is increasing in both developed and developing countries. The prostate cancer mortality rate varies among different regions 2. Central American holds the highest mortality rate at around 26%, followed by Western Europe at about 10%, and Asia countries report the lowest at 2.5%. Prostate cancer mortality is highly correlated with age (above 65), high body mass index, and high blood pressure 3.

Prostate cancer can be indolent or very aggressive, and the latter usually metastasis to other organs. Thus, most men diagnosed with prostate cancer do not die from it, but from secondary metastatic cancers, such as liver, lung, and bone cancers 4. The therapeutic options for local prostate cancer patients generally include surgical prostatectomy, radiation therapy, and systemic treatments 5. The systemic treatments comprise hormonal therapy, chemotherapy, immunotherapy, and targeted therapy 6. A prostate cancer patient may receive one type of medication or a combined type of medications. Abiraterone acetate, an inhibitor of androgen biosynthesis, is being widely used to treat metastatic prostate cancer 7. Abiraterone acetate therapy has been demonstrated to decrease mortality and prolong the overall survival rate of prostate cancer patients 8.

microRNAs (miRNAs) are known to play critical roles in almost all biological processes, such as cell growth, migration and invasion, metabolism, apoptosis, and differentiation 9. miRNAs generally downregulate target gene expression by binding to its mRNA and suppressing mRNA translation. miRNAs are reported to participate in the initiation, development, and progression of various types of tumors 10. MiRNAs can also be recognized as oncogenes or tumor suppressor genes based on their primary targets and biological functions. Dysregulation of miRNA profiles has been identified in all types of human cancers, and the utility of miRNAs in cancer diagnosis, progression, and response to treatments is gaining increasing attention 11,12.

In recent years, many miRNAs have been found to be involved in mediating the response of cancer cells to chemotherapeutic drugs 13. For instance, upregulation of miR-21 promotes doxorubicin/adriamycin (DOX)-resistance through targeting phosphatase and tensin homolog (PTEN) in breast cancer cells 14. MiR-200 family were reported to be involved in regulating taxanes sensitivity in ovarian cancer cells. In addition, downregulated miR-148a was identified to be responsible for enhanced paclitaxel resistance in hormone-refractory prostate cancer cells (PC3 and DU145) 15. A better comprehension of miRNAs in cancer drug response will enable the intervention of new therapeutic miRNA-related methods for prostate cancer patients 16.

In this study, we aimed to reveal the potential miRNA that participated in the regulation of the sensitivity to abiraterone acetate in prostate cancer cells. Through miRNA microarray analysis, we identified miR-143 as the most significantly downregulated miRNA among 25 miRNAs in PC3-AbiR cells compared to PC3, suggesting miR-143 might play a critical role in the regulation of abiraterone acetate sensitivity of prostate cancer cells. Hence, we forced on investigating the role of miR-143 in controlling prostate cancer cells' susceptibility to abiraterone acetate.

## Materials and Methods

### Cell lines

PC3 cell line was purchased from American Type Culture Collection (ATCC, Manassas, VA, USA), and was cultured in RPMI-1640 medium (Invitrogen, Waltham, MA, USA) supplied with 10% fetal bovine serum (FBS, Gibco, Grand Island, NY, USA). To establish abiraterone acetate resistance cell line, PC3 cells were maintained in the increased concentrations of abiraterone acetate (1 μM ~ 20 μM) over 12 months. The remaining PC3 cells are able to survive under abiraterone acetate treatment (20 μM), and were referred to as PC3-AbiR cells. The PC3-AbiR cells were maintained in medium with abiraterone acetate (10 μM, Selleck, Houston, TX, USA).

### Establishment of miR-143 stable expressing cell lines

Lentivirus carrying miR-143 (LPP-HmiR0084-MR03-050-S) or miR-control (LP606-050) were purchased from GeneCopoeia (Rockville, MD, USA). PC3-AbiR cells were transduced with miR-143 or miR-control lentivirus at MOI=5. After transduction, the infected PC3-AbiR cells were undergoing two rounds of puromycin selection. The survived PC3-AbiR cells were referred to PC3-AbiR-miR-143 and PC3-AbiR-miR-control.

### Quantitative real-time PCR (qRT-PCR)

Total RNA from decidua was isolated using TRIzol reagent (ThermoFisher Scientific, Waltham, MA, USA). 1 μg RNA was synthesized into cDNA using High-Capacity RNA-to-cDNA™ Kit (ThermoFisher Scientific). qRT-PCR amplification was performed on a LightCycler® 96 real-time PCR system (Roche, Shanghai, China) using SYBR™ Green PCR Master Mix (ThermoFisher Scientific) with cDNA and primers.

### Western blot

Protein samples were loaded on an 8% precast protein gels, and separated by SDS-PAGE. Subsequently, the separated proteins on the gel were transferred onto a polyvinylidene difluoride membrane. After blocked using a blocking buffer, the membrane was immersed in blocking buffer containing primary antibodies overnight. After incubation with secondary antibodies, the membrane was soaked in enhanced chemiluminescence. The protein signals were captured by a BioRad imaging system (ChemiDoc XRS+). GAPDH was used as a control for normalization. Antibodies against p-Bcl-2, Bcl-2, p-JNK, JNK, and GAPDH were purchased from Abcam (Pudong New District, Shanghai, China).

### Cell Counting Kit-8 (CCK-8) assay

PC3-AbiR-miR-143 and PC3-AbiR-miR-control were treated with or without abiraterone acetate for 48 hours. Cell viability was assessed using CCK-8 assay (ab228554, Abcam) following manufactory's instruction.

### Flow cytometry

PC3-AbiR-miR-143 and PC3-AbiR-miR-control were treated with or without abiraterone acetate for 48 hours. Cells were stained with Annexin V labeled with CF Blue and propidium iodide (PI, Sigma, St. Louis, MO, USA). After incubated in the dark room for 15 mins, cells were washed twice with cold phosphate-buffered saline (PBS). Apoptotic cell population was assess using BD Accuri™ C6 Plus Flow Cytometer.

### Animal experiments

Male BALB/c nude mice were purchased from the Vital River Laboratory Animal Technology Co. Ltd. (Beijing, China). 2×10^6^ PC3-AbiR-miR-143 or PC3-AbiR-miR-control cells were mixed with Matrigel (BD, USA) and 100 μl tumor cell mixture were subcutaneously inoculated into the flanks of each mouse. After the tumor volume reached 50-100 mm3, PC3-AbiR-miR-143 or PC3-AbiR-miR-control tumor bearing mice were received oral administration of vehicle or abiraterone acetate (200 mg/kg). Tumor volume was measured at three days interval by formulation of Volume=1/2×length×width^2^. Mice were sacrificed 21 days post-tumor implantation. Tumor tissue from each mouse was collected and weighted. This study was performed in strict accordance with the NIH guidelines for the care and use of laboratory animals (NIH Publication No. 85-23 Rev. 1985). The animal experiment protocol has been approved by Ethics Committee of the LONGHUA Hospital Shanghai University of Traditional Chinese Medicine.

### Immunohistochemistry staining

Tumor tissue sections were sequentially undergoing dewaxing, rehydration, and H_2_O_2_ treatment. Antigen retrieval was performed by immersing tissue sections in a container with antigen retrieval buffer (Tris/EDTA pH 9.0, sodium citrate pH 6.0) and boiling the container in a pressure cooker for 3 mins at its full pressure. After that, the tissue sections were covered with blocking buffer for 60 mins at room temperature, followed by incubation with Anti-Ki67 antibody [SP6] (ab16667, Abcam) overnight in cold room. Tissue sections were washed and incubated with secondary antibody. The slides were developed using the diaminobenzidine substrate kit following manufacture's instruction.

### Statistical analysis

All data are presented as means ± standard deviation (SD). Dots in the plot figures represent the biological replicates. Difference was assessed by Student's t-test between two groups, ANOVA analysis with a post hoc test between multiple groups. P value less than 0.5 was considered as statistically significant.

## Results

### Downregulation of miR-143 in abiraterone acetate resistance prostate cancer cell line

**Figure 1:**
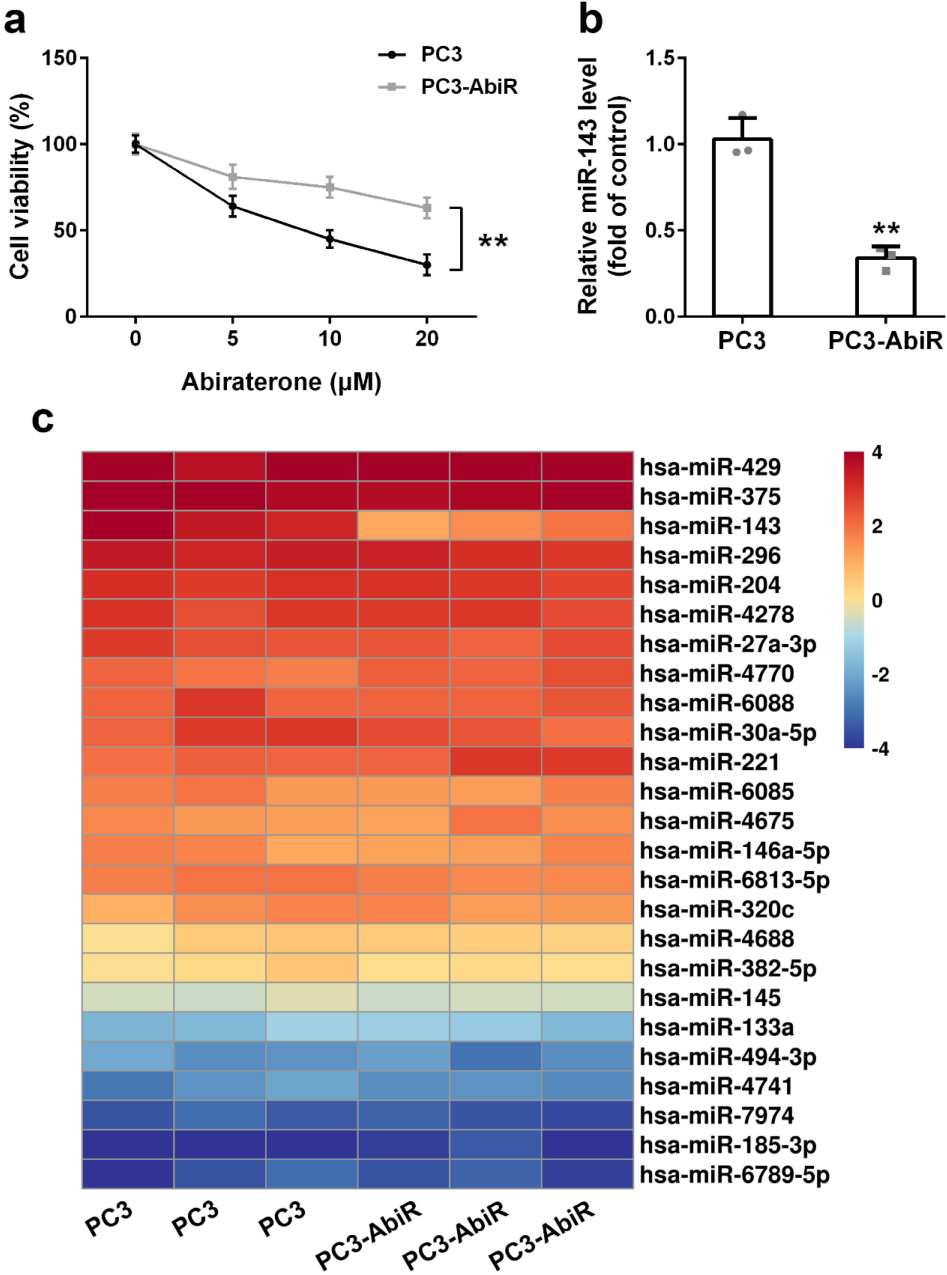
miR-143 is downregulated in abiraterone acetate resistance prostate cancer. (a) PC3 and PC3-AbiR cells were treated with different concentrations of abiraterone acetate for 48 hours, cell viability was calculated. (b) miR-143 levels of PC3 and PC3-AbiR were analyzed by qRT-PCR. (c) Relative expression levels of miRNAs in PC3 and PC3-AbiR were checked by microarray. Data were presented as mean±SD. n=3. Student's t test was used to evaluate the statistical significance. **p< 0.01.

PC3 and PC3-AbiR cells were treated with different concentrations of abiraterone acetate for 48 hours, and the cell viability was determined by CCK-8 assay. The results showed that abiraterone acetate treatment resulted in significantly more cell death in PC3 than PC3-AbiR, confirming PC3-AbiR cells are more resistant to abiraterone acetate than PC3 cells (Figure 1a).

To explore which miRNA might be involved in the regulation of abiraterone acetate sensitivity in PC3 cells, a miRNA microarray assay including 25 miRNAs was performed on PC3 and PC3-AbiR cells. As depicted in Figure 1b, among all analyzed 25 miRNAs, miR-143 was the most significantly downregulated miRNAs in PC3-AbiR cells compared to PC3 cells (Figure 1c). Downregulation of miR-143 in PC3-AbiR cells was further confirmed by RT-qPCR (Figure 1b). Thus, miR-143 was chosen for further analysis.

### miR-143 enhanced the sensitivity of PC3-AbiR cells to abiraterone acetate in vitro

**Figure 2:**
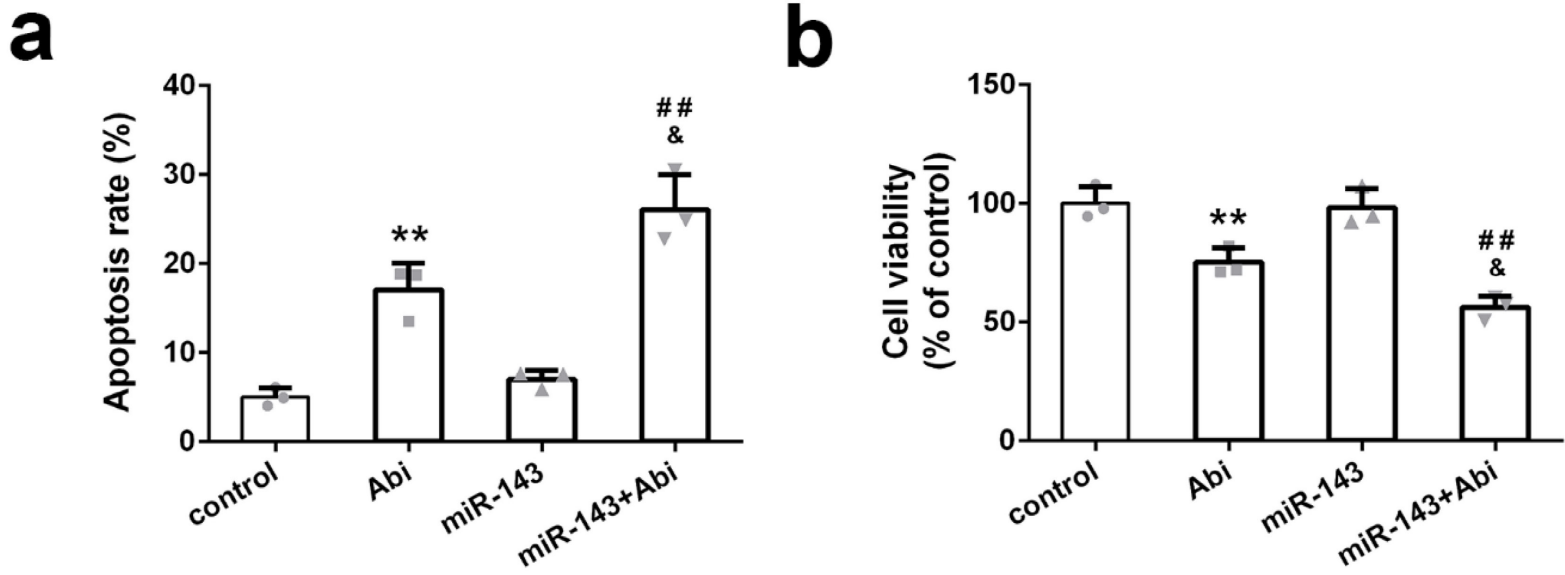
miR-143 reduced abiraterone acetate resistance ability of PC3-AbiR cells. (a) The apoptotic cell death of PC3-AbiR cells was analyzed by FACS analysis and apoptosis rate was calculated. (b) Cell viability of PC3-AbiR cells in each group was detected by CCK8 kits. Data were presented as mean±SD. n=3. One-way ANOVA followed by Tukey post-hoc test was used to evaluate the statistical significance. **p< 0.01 compared with control group; &p<0.05 compared with Abi group; ##p<0.01 compared with miR-143 mimics group.

PC3-AbiR cells were transfected with miR-Control or miR-143 for 48 hours and then treated with or without abiraterone acetate for 48 hours. The cell apoptosis and cell viability were measured by PI-Annexin V staining and CCK8 kit, respectively. The results illustrated that abiraterone acetate treatment induced markedly more apoptosis and cell death in PC3-AbiR cells with miR-143 overexpression compared to PC3-AbiR cells with miR-Control overexpression (Figure 2a-2b). The apoptosis rate and cell death rate were comparable between PC3-AbiR-miR-Control and PC3-AbiR-miR-143 without abiraterone acetate (Figure 2a-2b). These results suggested that miR-143 overexpression enhanced abiraterone acetate sensitivity in PC3-AbiR cells.

### JNK is a potential target of miR-143

**Figure 3:**
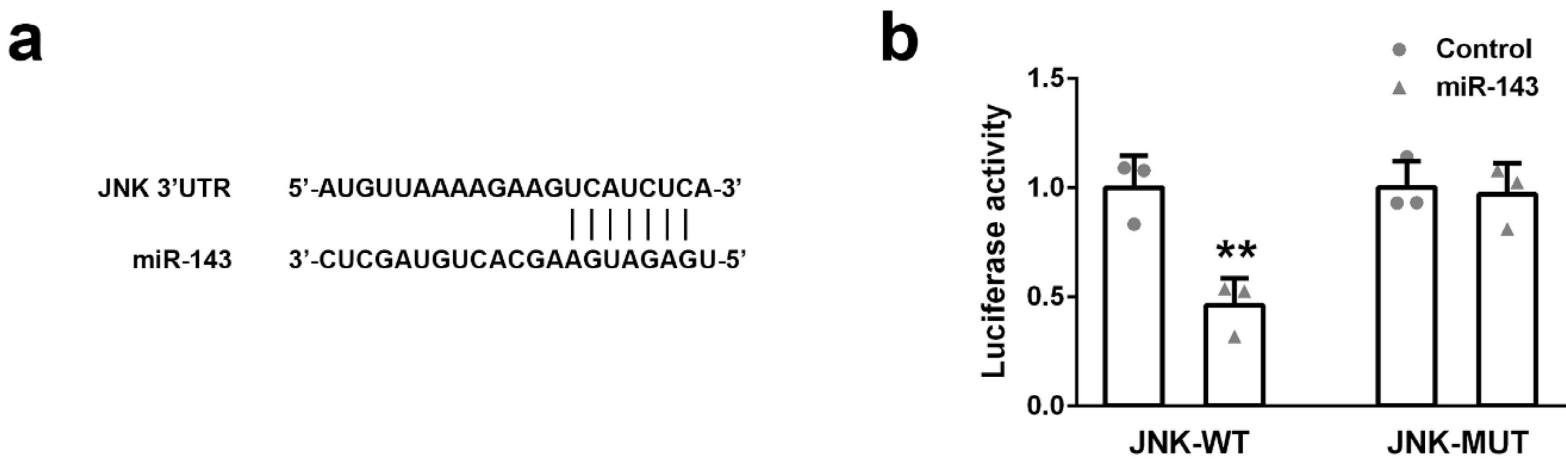
The target gene of miR-143 were predicted and validated. (a) The binding sites of miR-143 and JNK 3'UTR were predicted by the website Target Scan. (b) Dual-luciferase reporter assays were performed in PC3 cells. Data were presented as mean±SD. n=3. Student's t test was used to evaluate the statistical significance. **p< 0.01 compared with control group.

To identify the potential target of miR-143, we found a sequence located in 3'UTR of JNK contains the binding site of miR-143, implying JNK might be a potential target of miR-143 (Figure 3a). To prove this hypothesis, a DNA sequence of 3'UTR of JNK containing either wild-type (JNK-WT) or mutant (JNK-MUT) miR-143 binding site was cloned into the dual-luciferase reporter plasmid. Subsequently, the plasmid (JNK-WT or JNK-MUT) was co-transfected with miR-Control or miR-143 into PC3 cells. The luciferase activity was measured 48 hours after transfection. As showed in Figure 3b, overexpression of miR-143 specifically suppressed the luciferase activity of the plasmid containing the wild-type, but not mutant, miR-143 binding site in the 3'UTR of JNK (Figure 3b). This evidence suggested that JUK is a direct target of miR-143.

### miR-143 regulated p-JNK and p-Bcl2 but not JNK and Bcl2

**Figure 4:**
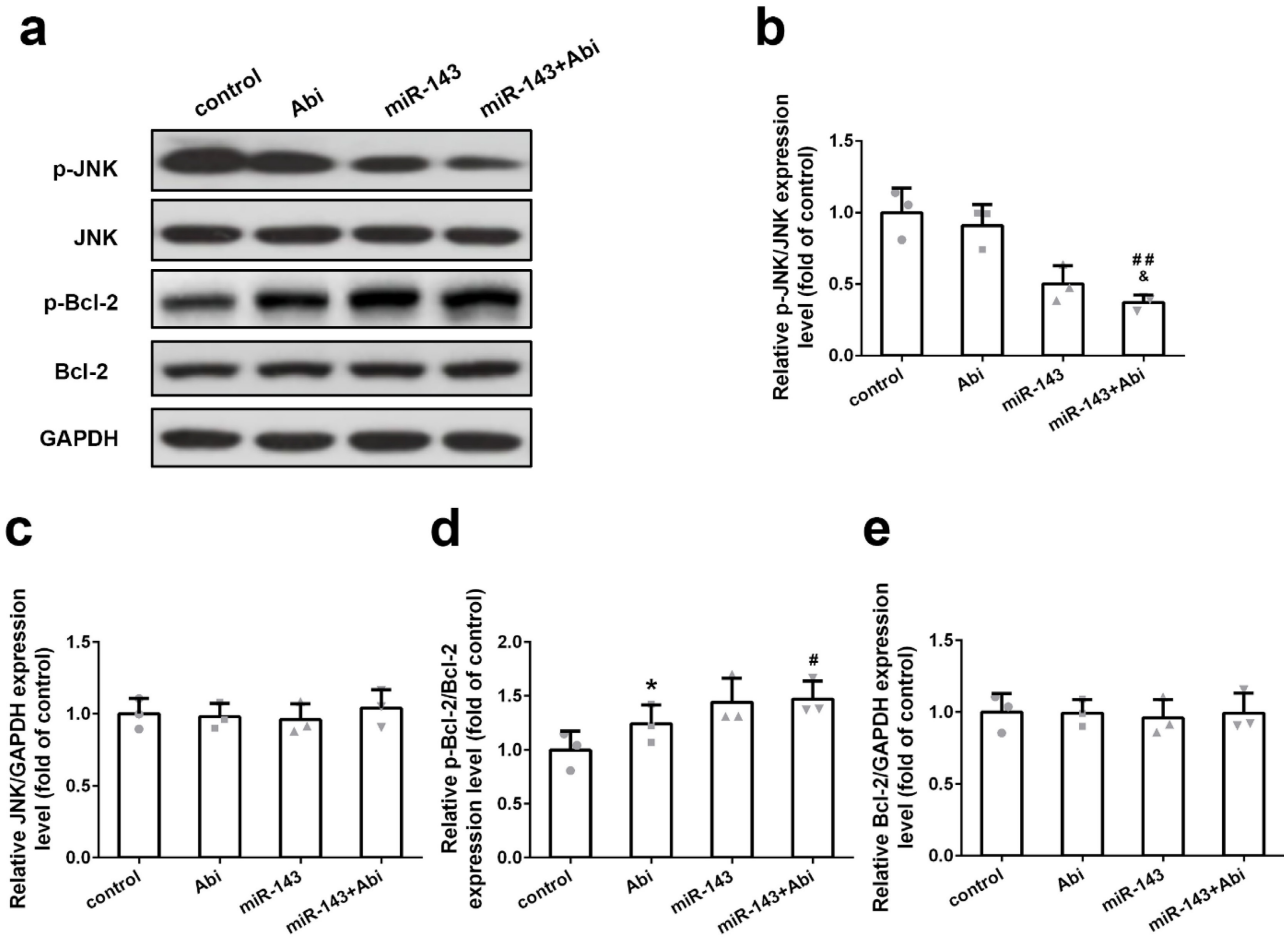
Effect of miR‐143 upregulation on JNK/Bcl-2 signaling pathway in PC3- AbiR. Western blot was used to detect protein levels (a) and relative protein expression levels of p-JNK (b), JNK (c), p-Bcl-2 (d) and Bcl-2 were analyzed. Data were presented as mean±SD. n=3 for each group. One-way ANOVA followed by Tukey post-hoc test was used to evaluate the statistical significance. *p< 0.05 compared with control group; #p<0.05, ##p<0.01 compared with miR-143 mimics group; &p<0.05 compared with Abi group.

To further study the effect of miR-143 on target gene expression, PC3-AbiR cells were transfected with miR-Control or miR-143, and then treated with or without abiraterone acetate. The expression levels of p-JNK, JNK, p-Bcl-2, and Bcl-2 were tested by western blot assay. As demonstrated in Figure 4a-e, overexpression of miR-143 significantly decreased p-JNK levels and enhanced p-Bcl2 levels in PC3-AbiR cells. The combination of miR-143 and abiraterone acetate exerted most profound effects on reducing p-JNK and increasing p-Bcl2. Of note, overexpression of miR-143 had negligible effects on total JNK and Bcl-2 expression (Figure 4a-4e). Furthermore, blocking miR-143 expression using miR-143 inhibitor abolished the effect of miR-143 on the expression of p-JNK and p-Bcl-2 (Figure S1).

### miR-143 promoted the sensitivity of PC3-AbiR tumor to abiraterone acetate in vivo

**Figure 5:**
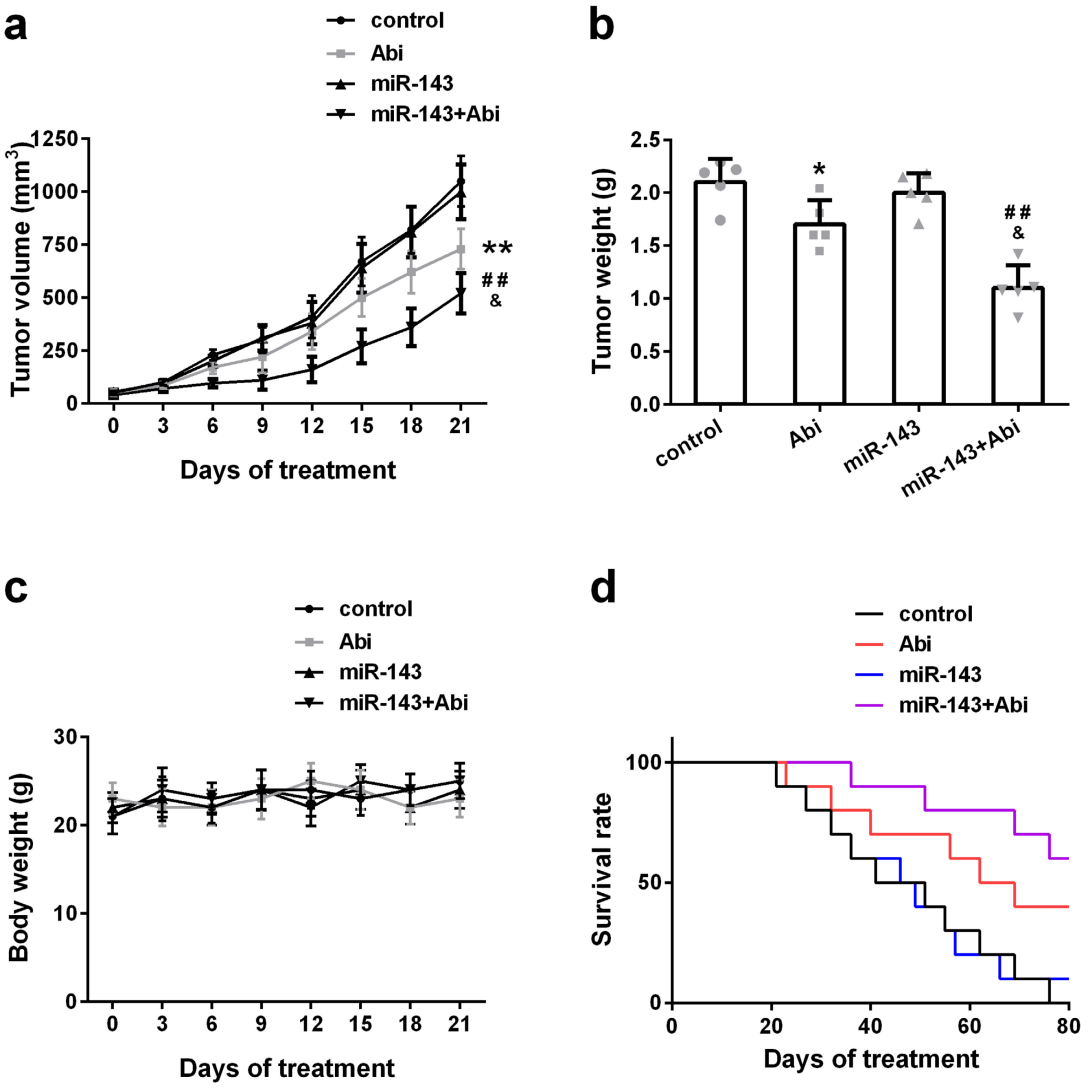
miR‐143 enhanced abiraterone acetate treatment in mice. The volume (a) and weight (b) of subcutaneous xenografts in each group were analyzed. The body weight (c) and survival rate (d) of mice were recorded. Data were presented as mean±SD. n=5 for each group. One-way ANOVA followed by Tukey post-hoc test was used to evaluate the statistical significance for panel b, and two-way ANOVA followed by Bonferroni post hoc test for panels a and c. *p< 0.05, **p< 0.01 compared with control group; ##p<0.01 compared with miR-143 mimics group; &p<0.05 compared with Abi group.

To investigate whether overexpression of miR-143 could enhance the anti-tumor effect of abiraterone acetate on prostate cancer cells, PC3-AbiR cells were transduced with lentivirus expressing miR-Control or miR-143. After two round puromycin selection, PC3-AbiR stably expressing miR-Control or miR-143 were established. Two cell lines (control, miR-143) were subcutaneously injected into nude mice, and the tumor-bearing mice received oral administration of abiraterone acetate at 200 mg/kg or PBS as a control. We observed that the PC3-AbiR tumor treated with abiraterone acetate grew significantly slower than PC3-AbiR-control, and the tumor-bearing mouse survival rate of PC3-AbiR with abiraterone acetate was also evidently higher than PC3-AbiR-control (Figure 5a-5d). Overexpression of miR-143 had no effect on tumor growth and mouse survival rate. On the contrary, forced miR-143 expression combined with abiraterone acetate treatment substantially reduced the tumor growth and prolonged the mouse survival rate (Figure 5a-5d). Taken together, these results overexpression of miR-143 could enhance the anti-tumor effect of abiraterone acetate on PC3-AbiR cells. Of note, the mouse body weight was comparable among four groups and did not change significantly before and after treatment, implying miR-143 and/or abiraterone acetate were safe to mice (Figure 5c).

### Ki67 was lowest in PC3-AbiR tumor with miR-143 overexpression and abiraterone acetate treatment

**Figure 6:**
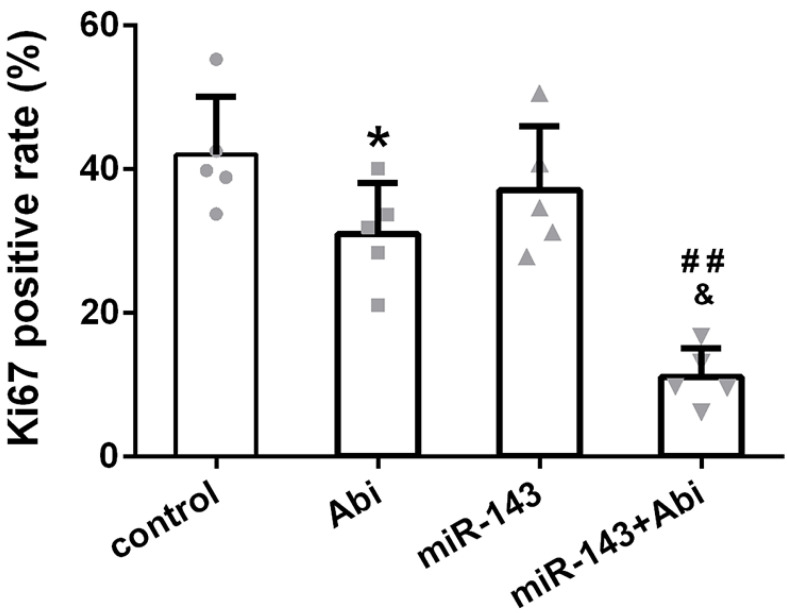
The percentage of Ki67-positive cells in different experimental groups. Data were presented as mean±SD. n=5 for each group. One-way ANOVA followed by Tukey post-hoc test was used to evaluate the statistical significance. *p< 0.05 compared with control group; ##p<0.01 compared with miR-143 mimics group; &p<0.05 compared with Abi group.

The expression of Ki67, a cell proliferation marker, was stained in tumor tissues from four groups. The IHC results revealed that the Ki67 positive rate was comparable between Control tumor tissues and miR-143 overexpressing tumor tissues. The Ki67 positive rate was decreased under abiraterone acetate treatment and was lowest in abiraterone acetate+miR-143 tumor tissues among four groups, confirming that miR-143 promotes tumor inhibition effect of abiraterone acetate on PC3-AbiR cells (Figure 6).

## Discussion

Numerous studies have proved that miR-143 is a universal anti-oncomiR 17. Downregulation of miR-143 was described in various cancer cell lines and tumor tissues, including prostate, breast, gastric, ovarian, colon, bladder, and liver cancer, neuroblastoma, renal cell carcinoma, and osteosarcoma 18,19. Decreased miR-143 levels are also correlated with the presence of metastases in many human cancers, such as prostate, breast, and cervical cancer, glioma, and hepatocellular carcinoma 17,19. Furthermore, lower expression levels of miR-143 are reported to be correlated with poorer prognosis and shorter overall survival rate in multiple types of cancer patients 19,20.

Accumulating evidence has revealed that miR-143 can enhance the drug sensitivity of various human cancer cell lines 21. For example, Borralho et al. reported that overexpression of miR-143 decreased cell proliferation and increased cell death in colorectal cancer cells. miR-143 restoration increased the 5-fluorouracil sensitivity of colon cancer cells 22. Similarly, Xu et al. found miR-143 downregulation in prostate cancer cells, and miR-143 overexpression suppressed cell proliferation and enhanced prostate cancer cell sensitivity to docetaxel by targeting KRAS 23. Interestingly, Ma et al. reported that miR-143 upregulation is capable of inducing the apoptosis of LNCap, a prostate cancer cell line, by targeting BCL-2 expression 24. Consistently with their findings, we also found that overexpression of miR-143 enhanced abiraterone acetate-induced cell death on PC3-AbiR cells.

To explore the underlying molecular mechanism, we identified JNK as a direct target of miR-143 by using luciferase report assay and Western blot assay. Of note, Bcl-2 has been confirmed as a direct target of miR-143 in colon, ovarian, bladder, and cervical cancer 24-26. In contrast, miR-143 had no effect on Bcl-2 expression in PC3-AbiR cells. Although miRNAs that regulate target gene expression might be cellular- or tissue-specific, the reason why miR-143 cannot regulate JNK and Bcl-2 expression in PC3-AbiR cells remains elusive.

Importantly, we found that overexpression of miR-143 decreased p-JNK levels and enhanced p-Bcl-2 levels. The detailed mechanism requires further investigation. Because KRAS and AKT are two direct targets of miR-143 in multiple cancers, and KRAS and AKT are the upstream regulators of JNK/Bcl-2 signaling pathway 19,27. We hypothesized that miR-143 might mediate JNK/Bcl-2 signaling pathway through targeting KRAS and/or AKT in PC3-AbiR. Further studies are needed to prove this hypothesis.

Finally, we further confirmed that miR-143 can enhance abiraterone acetate sensitivity of PC3-AbiR to abiraterone acetate using a PC3-AbiR tumor bearing mouse model, and the results were consistent with these findings using a cellular model.

Although this study provided some direct evidence that miR-143 downregulation may be a contributing factor in the development of abiraterone acetate-resistant prostate cancer cells, there are some limitations that need to be addressed further. For instance, in addition to PC-3 and PC3-AbiR cell lines, at least another set of abiraterone acetate sensitive and resistant prostate cancer cell lines is highly desirable in the following study. Surgically resected human surgical abiraterone acetate sensitive and resistant prostate cancer samples are strongly required in order to confirm the finding of downregulation of miR-143 in abiraterone acetate resistant prostate cancer samples when compared to abiraterone acetate sensitive prostate cancer samples.

## Conclusion

Collectively, we are the first to reveal that miR-143 plays a positive role in promoting abiraterone acetate-resistant prostate cancer cells to abiraterone acetate treatment-induced apoptosis in vitro and in vivo. Restoration of miR-143 may be beneficial for developing a new method to treat abiraterone acetate- resistant prostate cancer patients.

## Supplementary Material

Supplementary figure.
