# Supplementary material for: miR-143 mediates abiraterone acetate resistance by regulating the JNK/Bcl-2 signaling pathway in prostate cancer
Source: J Cancer. 2022 Nov 21;13(15):3652–9. doi: 10.7150/jca.78246 (PMC9809307; doi:10.7150/jca.78246)
Supplement: Supplementary file 1 — Supplementary figure. [file jcav13p3652s1.pdf]

Supplementary materials

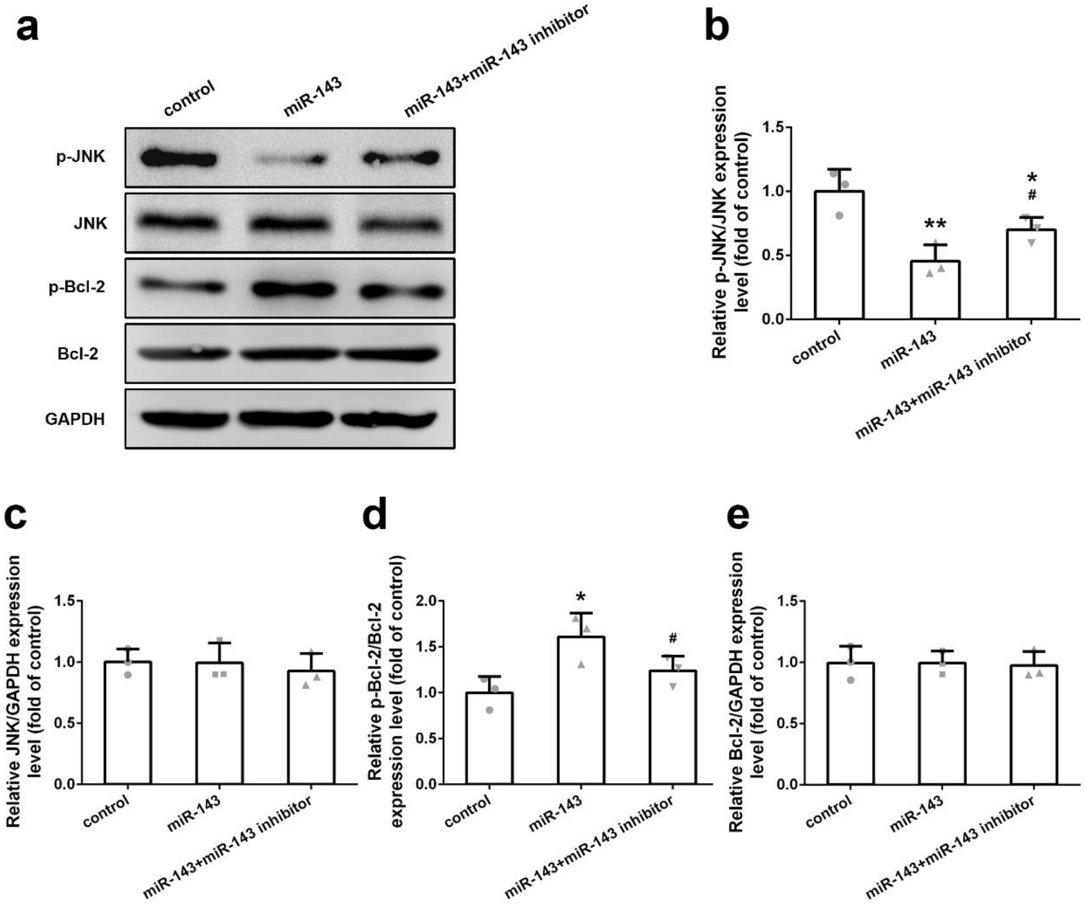

Figure S1. miR-143 inhibitor can reverse the expression of JNK/Bcl-2 induced by miR-143 upregulation in PC3- AbiR. Western blot was used to detect protein levels (a) and relative protein expression levels of p-JNK (b), JNK (c), p-Bcl-2 (d) and Bcl-2 were analyzed. Data were presented as mean±SD. n=3 for each group. One-way ANOVA followed by Tukey post-hoc test was used to evaluate the statistical significance. *p< 0.05, **p< 0.01 compared with control group; #p<0.05 compared with miR-143 group.
